# Dipeptidyl Peptidase-4 deficiency effectively protects the brain and neurological function in rodent after acute Hemorrhagic Stroke

**DOI:** 10.7150/ijbs.42677

**Published:** 2020-10-16

**Authors:** Hon-Kan Yip, Mel S. Lee, Yi-Chen Li, Pei-Lin Shao, John Y. Chiang, Pei-Hsun Sung, Chien-Hui Yang, Kuan-Hung Chen

**Affiliations:** 1 Division of Cardiology, Department of Internal Medicine, Kaohsiung Chang Gung Memorial Hospital and Chang Gung University College of Medicine, Kaohsiung 83301, Taiwan.; 2 Institute for Translational Research in Biomedicine, Kaohsiung Chang Gung Memorial Hospital, Kaohsiung 83301, Taiwan.; 3 Center for Shockwave Medicine and Tissue Engineering, Kaohsiung Chang Gung Memorial Hospital, Kaohsiung 83301, Taiwan.; 4 Department of Medical Research, China Medical University Hospital, China Medical University, Taichung 40402, Taiwan.; 5 Department of Nursing, Asia University, Taichung, 41354, Taiwan.; 6 Division of Cardiology, Department of Internal Medicine, Xiamen Chang Gung Hospital, Xiamen 361028, Fujian, China.; 7 Department of Orthopedics, Kaohsiung Chang Gung Memorial Hospital and Chang Gung University College of Medicine, Kaohsiung 83301, Taiwan.; 8 Department of Computer Science and Engineering, National Sun Yat-Sen University, Kaohsiung, Taiwan.; 9 Department of Anesthesiology, Kaohsiung Chang Gung Memorial Hospital and Chang Gung University College of Medicine, Kaohsiung 83301, Taiwan.

**Keywords:** acute hemorrhagic stroke, dipeptidyl peptidase-4 activity, brain infarct area, neurological function, inflammation, angiogenesis, oxidative stress

## Abstract

This study tested the hypothesis that abrogated dipeptidyl peptidase-4 (DPP4) activity played a crucial role on reducing stroke volume and preserving neurological function in rodent after acute hemorrhagic stroke (AHS). Animals (n=6/each group) were categorized into group 1 (sham-control of F344 rat), group 2 (sham-control of DPP4-deficiency rat), group 3 [AHS by right cerebral injection of autologous blood (100 µL) in F344 rat], group 4 (AHS + sitagliptin/600 mg/kg 3 h prior to and at 3 h then once per day after AHS) and group 5 (AHS in DPP4-deficiency rat). The results of corner test showed the neurological function was significantly improved from days 3, 7, and 14 in groups 4 and 5 than in group 3 (all *p*<0.001). By days 1 and 14 after AHS procedure, the circulating levels of SDF-1α and GLP-1 were significantly increased from groups 1/2 to group 5 (all *p*<0.001), whereas circulating DPP4 activity was significantly increased in group 3 than other groups (all *p*<0.001). The brain ischemic area (BIA) was highest in group 3, lowest in groups 1/2 and significantly lower in group 5 than in group 4 (all *p*<0.0001). The protein expressions of oxidative-stress/inflammatory/apoptotic/cell-proliferation signaling, and the cellular expressions of inflammatory/DNA-damaged biomarkers exhibited a similar pattern to BIA among the groups (all *p*<0.01). In conclusion, deprivation of DPP4 activity protected the brain from AHS damage and preserved neurological function.

## Introduction

Stroke, a growing epidemic, is still the leading cause of mortality, disability and dependence worldwide 1-4. Yet despite a better understanding of prevalence, etiology and stroke mechanisms, advancements in imaging leading to earlier and more accurate diagnosis and improved pharmacological agents; a universally accepted effective and safe strategic management for both ischemic stroke (IS) and hemorrhagic stroke (HS) patients remains undefined.

The HS accounts for nearby 30% of all types of acute stroke and usually has poorer prognostic outcomes as compared to those of IS. Additionally, acute HS (i.e., AHS) patients who required surgical intervention will frequently develop permanent disability 5-11. Furthermore, there is no data as to whether the pharmacomodulation is beneficial in patients who suffer from AHS and survive with permanent disability. Accordingly, finding a safe and effective therapeutic alternative for patients following acute HS, especially for those unsuitable for surgical intervention, is mandatory for physicians.

Previous studies have revealed that the glucagon-like peptide (GLP)-1, a hormonal peptide, can cross the blood-brain barrier 12 and mediate in the therapeutic actions of dipeptidyl peptidase (DPP)-IV (DPP4) inhibitors 13. Interestingly, DPP4 inhibitor, an oral hypoglycemic agent currently used for treating type 2 diabetic patients, has been found to be able to enhance circulating GLP-1 and stromal cell-derived factor (SDF)-1α levels through inhibition of DPP4 activity 14, 15 which, in turn, provides cardiovascular protective effect probably through the anti-inflammatory, anti-oxidative stress and anti-atherosclerotic actions of GLP-1 16.

Growing data from experimental studies have demonstrated that DPP4 inhibitors have capacity of neuroprotective effect in setting of acute IS 17-22. Additionally, our previous study has also demonstrated that sitagliptin (Sita) (i.e., a DPP4 inhibitor) therapy attenuated brain damage and cognitive impairment in mice with chronic cerebral hypo-perfusion through suppressing oxidative stress and inflammatory reaction 23. Being worthy of note, another our previous study 24 has displayed that Sita therapy protects rat kidneys from acute ischemia-reperfusion injury via upregulation of GLP-1 and GLP-1 receptors. Surprisingly, there is no report regarding the role of DPP4 inhibitor on protecting the brain damage against the HS.

In view of the ischemia, generation of inflammation, oxidative stress and reactive oxygen species (ROS) are the major contributors for progressive brain damage after AHS and, based on the aforementioned issues, it is praiseworthy to investigate whether DPP4 inhibitor therapy really limits the brain hemorrhagic volume and preserves neurological function, as well as improves prognostic outcome after AHS.

## Materials and Methods

### Ethics statement

All animal procedures were approved by the Institute of Animal Care and Use Committee at Kaohsiung Chang Gung Memorial Hospital (Affidavit of Approval of Animal Use Protocol No. 2016112402) and performed in accordance with the Guide for the Care and Use of Laboratory Animals.

Animals were housed in an Association for Assessment and Accreditation of Laboratory Animal Care International (AAALAC; Frederick, MD, USA)-approved animal facility in our hospital with controlled temperature and light cycles (24 °C and 12/12 light cycle).

### Procedure and protocol of AHS induction by injection of autologous blood into the brain ad animal grouping

Pathogen-free, adult male Fischer344 and DPP4-deficiency (DPP4^D^) rats weighing 300-320 g (Charles River Technology, BioLASCO Taiwan Co. Ltd., Taiwan) were utilized in the present study. The procedure and protocol were based on the previous report 25 with minimal modification. In detail, the stereotactic apparatus was first set up and animals were then anesthetized by inhalational 2.0% isoflurane, placed in a prone position on a warming pad at 37 °C for AHS induction. To ensure the depth of anesthesia, the rats were tested by the pinch. After loss of consciousness, the rats were fixed on the stereotactic frame using a nose clamp and two ear bars. After shaving the fur, the superior surface of rat head was prepared by sterile iodophor wipes as surgical field. A longitudinal incision was made on the surface of head to expose the bregma. Using the stereotactic apparatus, a left point 3 mm lateral to the bregma of the coronal suture was marked. A burr hole through the skull (1 mm) was drilled at the marked point by a high-speed stereotaxic drill. Then, 100 μl autologous blood was injected into corpus striatum at a rate of 10 μl/min through a 26-gauge needle (coordinates: 0.2 mm anterior, 5.5 mm ventral, and 3.5 mm lateral to bregma) with the use of a micro-infusion pump (Harvard Apparatus Inc). The sham control rats only received a needle insertion. The syringe remained at the place about 7 min after the injection was completed and was then slowly removed. Once the syringe was removed, sterile bone wax was used to plug the hole quickly. The skin on the surface of head was then closed by using 4-0 Monocryl. The rats were finally removed from the stereotactic apparatus and were allowed to recover from anaesthesia in a portable animal intensive care unit (ThermoCare**^®^**) with free access to food and water for 24 hours.

Animals (n=6 in each group) were categorized into group 1 [sham-control of F344 rat (i.e., F344^SC^)], group 2 [sham-control of DPP4^D^ rat (i.e., DPP4^D-SC^)], group 3 [AHS by right cerebral injection of autologous blood (100 μL) in F344 rat (i.e., F344^HS^)], group 4 (F344^HS^ + Sita/600 mg/kg 3h prior to and at 3h, followed per day after AHS, orally) and group 5 [AHS in DPP4^D^ rat (i.e., DPP4^D-HS^)].

### Inclined plane test of hind limb muscle power and coordination

To assess the muscle power and coordination of the hind limbs of the rats, an inclined plane test was adopted as previously described with slight modifications 26. In detail, during a five-day period of acclimatization in a temperature- and humidity-controlled room with 12-hour light-dark cycle and free access to water and standard animal chow, the rats were gently handled by laboratory personnel five times a day to let them be accustomed to human manipulation. In the following three days of training, the animals were placed on an inclined plane made of cardboard on which a horizontal friction trip provided a foothold for the animal's hind limbs as the inclination angle increased to prevent the animal from sliding down the slope. During the actual inclined plane test, each animal was placed on the inclined plane so that a secure foothold was established between the claws of its hind limbs and the friction trip. After confirmation of correct body position in the absence of anxious behavior and abnormally tense muscle tone of the animal, the inclination angle was slowly increased till the animal's hind limbs lost grasp of the friction trip and slid down the plane. The inclination angle was then recorded. After performing the experiment three times for each animal, the mean inclination angle was obtained by averaging the three recordings. The whole procedure was conducted by two independent technicians blinded to grouping of the animals.

### Brain magnetic resonance imaging (MRI) examination for assessment of brain ischemic volume (BIV) in acute phase of brain hemorrhagic stroke

The procedure and protocol for brain magnetic resonance imaging (MRI) have been described in our previous study 27. MRI was assessed at days 2 and 7 after ASH induction. Briefly, for MRI, rats were anesthetized by 3% inhalational isoflurane with room air and placed in an MRI-compatible holder (Biospec 94/20, Bruker, Ettingen, Germany). MRI data were collected using a Varian 9.4T animal scanner (Biospec 94/20, Bruker, Ettingen, Germany) with a rat surface array. The MRI protocol consisted of 40 T2-weighted images. Forty continuous slice locations were imaged with a field-of-view of 30 mm x 30 mm, an acquisition matrix dimension of 256 x 256 and slice thickness of 0.5 mm. The repetition time (TR) and echo time (TE) for each fast spin-echo volume were 4200 ms and 30 ms, respectively. Custom software, ImageJ (1.43i, NIH, USA), was used to process the region of interest (ROI). Planimetric measurements of images from MRI T2 were performed to calculate stroke volumes. Four of 6 animals in each group were randomly selected for brain MRI examination.

### Flow cytometric quantification of EPCs

The procedure and protocol of flow cytometry have been described in detail in our previous reports 27, 28. Briefly, venous route was adopted for blood sampling at different time points (at baseline and on days 1 and 14 after ASH induction). After treatment with red blood cell-lysing buffer, the cells remained were labeled with appropriate antibodies. Flow cytometric analysis for identification of cell surface markers was performed. The cells were immunostained for 30 minutes with monoclonal antibodies against primary antibodies, including CD 31, C-kit, KDR, Sca-1, CXCR4, vascular endothelial (VE) Cadherin and CD34. Secondary detection was performed using appropriate Alexa Fluor 488 (Molecular Probes, Eugene, OR, USA). Isotype-identical antibodies (IgG) served as controls. Flow cytometric analyses were performed by utilizing a fluorescence-activated cell sorter (Beckman Coulter FC500 flow cytometer).

### Measurement of the brain ischemic area (BHA)

For this investigation, additional four rats in each group were utilized. By the end of the study period, the rats were euthanized in each group and the brains were harvested and well prepared. Finally, three sections of H.E., stained brain specimen from each rat, were used and three randomly selected high-power fields (HPFs) (200×) were analyzed in each section after H.E stain. The mean number per HPF for each animal was then determined by summation of all numbers at each level divided by 9. Finally, the mean of the BIA was obtained.

### Immunofluorescent (IF) staining

The procedure and protocol for IF staining have been reported in our previous studies 23, 24, 27, 28. For IF staining, rehydrated paraffin sections were first treated with 3% H_2_O_2_ for 30 minutes and incubated with Immuno-Block reagent (BioSB, Santa Barbara, CA, USA) for 30 minutes at room temperature. Sections were then incubated with primary antibodies specifically against F4/80 (1:100, Santa Cruz Biotechnology), CD14 (1:200, Proteintech), γ-H2AX (1:500, Abcam), p53BP1(1:300, Novus Biologicals) glial fibrillary acidic protein (GFAP) (1:500, Dako), CD31 (1:100, Bio-Rad), von Willebrand factor (vWF) (1:500, Merck Millipore), stromal cell-derived growth factor (SDF)-1α (1:100, Santa Cruz Biotechnology) and CXCR4 (1:200, Abcam), while sections incubated with the use of irrelevant antibodies served as controls. Three sections of kidney specimen from each rat were analyzed. For quantification, three random HPFs (200× or 400× for ICH and IF studies) were analyzed in each section. The mean number of positively stained cells per HPF for each animal was then determined by summation of all numbers divided by 9.

### Western blot analysis

The procedure and protocol for Western blot analysis have been reported by our previous studies 23, 24, 27, 28. Briefly, equal amounts (50 μg) of protein extracts were loaded and separated by SDS-PAGE using acrylamide gradients. After electrophoresis, the separated proteins were transferred electrophoretically to a polyvinylidene difluoride membrane (GE, UK). Nonspecific sites were blocked by incubation of the membrane in blocking buffer [5% nonfat dry milk in T-TBS (TBS containing 0.05% Tween 20)] overnight. The membranes were incubated with appropriate primary antibodies [cleaved caspase 3 (1: 1000, Cell Signaling), cleaved poly (ADP-ribose) polymerase (PARP) (1: 1000, Cell Signaling), mitochondrial Bax (1: 1000, Abcam), nuclear factor (NF)-κB (1: 1000, Abcam), tumor necrosis factor (TNF)-α (1: 1000, Cell Signaling), interleukin (IL)-1β (1: 1000, Cell Signaling), inducible nitric oxide synthase (iNOS) (1: 250, Abcam), matrix metalloproteinase (MMP)-2 (1: 1000, Cell Signaling), MMP-9 (1: 1000, Abcam), tissue inhibitors of matrix metalloproteinase TIMP 1(1: 1000, Abcam), TIMP 2 (1: 1000, Abcam), NOX-1 (1: 1500, Sigma), NOX-2 (1: 1000, Sigma), γ-H2AX (1: 1000, Cell Signaling), phosphorylated (p)-Smad3 (1: 1000, Cell Signaling), transforming growth factor (TGF)-ß (1: 1000, Abcam), I-κB (1: 1000, Abcam), protein expression of Nuclear factor erythroid 2-related factor 2 (Nrf2) (1: 1000, Abcam), glucagon-like peptide (GLP)-1, SDF-1α (1: 1000, Cell Signaling), PI3K (1: 1000, Cell Signaling), phosphorylated (p)-Akt (1: 1000, Cell Signaling) and Actin (1: 1000, Millipore)] for 1 hour at room temperature. Horseradish peroxidase-conjugated anti-rabbit immunoglobulin IgG (1:2000, Cell Signaling, Danvers, MA, USA) was used as a secondary antibody for one-hour incubation at room temperature. The washing procedure was repeated eight times within one hour. Immunoreactive bands were identified by enhanced chemiluminescence (ECL; Amersham Biosciences, Amersham, UK) and exposed to Biomax L film (Kodak, Rochester, NY, USA). For quantification, ECL signals were digitized using Labwork software (UVP, Waltham, MA, USA).

### Analysis of circulating levels of SDF-1α and GLP-1 and DPP4 activity

Blood samples were drawn from rats in each group and stored at -80 °C until analyses of TNF-α, GLP-1 and DPP4 were performed in batches at the end of experiment. The serum concentrations of these parameters were examined in duplicate with a commercially available ELISA kit (R&D Systems, Minneapolis, MN, USA). Intra-individual variabilities in TNF-α, GLP-1 and DPP4 levels were assessed in each group with the mean intra-assay coefficient of variance < 1.8%.

### Procedure and protocol of cell culture

Neuro-2a (N2a: neuroblastoma cell line) was maintained in MEM medium supplemented with 10% fetal bovine serum and 1% PS, 2 mM L-glutamine, 0.1 mM non-essential amino acids, 1.0 mM sodium pyruvate, 100 U /ml penicillin G and 100 μg /ml streptomycin. During the cell culture, sitagliptin (50 ug/mL), H_2_O_2_ (50 uM) LY-294002 (1 uM) (i.e., an PI3K inhibitor) were added for MTT assay, Western blot and flow cytometric analysis of apoptosis (i.e., Annexin V study).

### Statistical analysis

Quantitative data were expressed as means ± SD. Statistical analysis was adequately performed by ANOVA followed by Bonferroni multiple comparison post hoc test. SAS statistical software for Windows version 8.2 (SAS institute, Cary, NC, USA) was utilized. A probability value <0.05 was considered statistically significant.

## Results

### Time courses of circulating levels of SDF-1α, GLP-1 and ECPs, and circulating DPP4 activity after AHS in animals measured by ELISA

**Figure 1:**
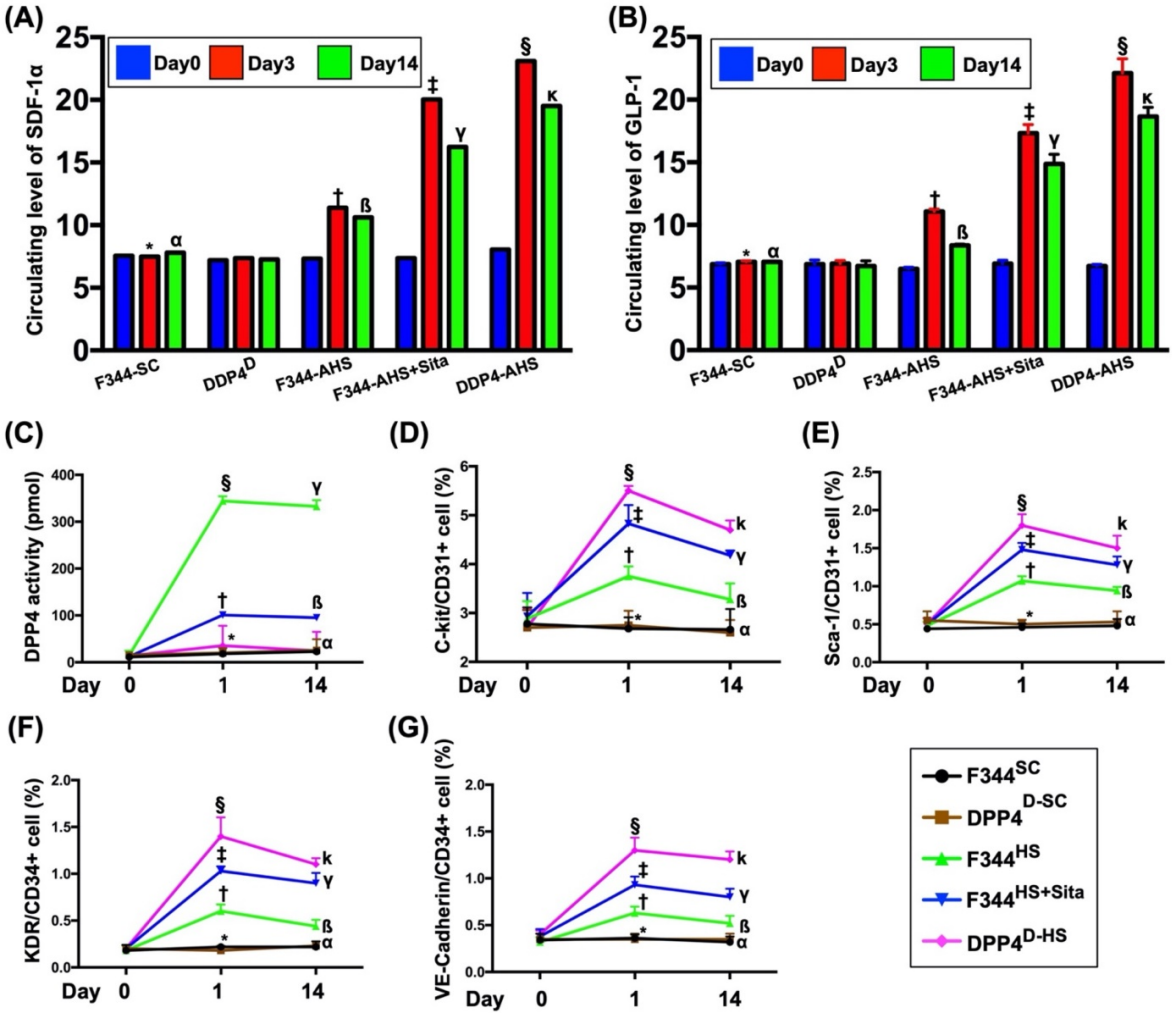
** Time courses of circulating levels of SDF-1α and GLP-1 after AHS in animals measured by ELISA.** (**A and B**) Illustrating the circulating levels of stromal cell-derived factor (SDF)-1α (pg/ml) (A) and glucagon-like peptide-1 (GLP-1) (pg/ml) at different time points. (1) By day 0 (blue bar chart) prior to AHS procedure, the circulating levels of SDF-1α and GLP-1 did not differ among the four groups, *p*>0.5. (2) By days 3 (red bar chart) and 14 (green bar chart) after AHS procedure, the circulating levels of SDF-1α and GLP-1 were significantly progressively increased from F344^SC^/DPP4^D-SC^ groups to DPP4^D-HS^ group; for day 3: * vs. other groups with different symbols (†, ‡, §), *p*<0.0001, for day 14: α vs. other groups with different symbols (ß, γ, κ), *p*<0.0001. (**C**) Illustrating the ELISA analysis of time courses of DPP4 activity in circulation. By day 0, *p* >0.5. By day 3, * vs. other groups with different symbols (†, ‡), p<0.0001. By day 14, α vs. other groups with different symbols (ß, γ), *p*<0.0001. (**D**) Illustrating the flow cytometric analysis of time courses of circulating number of C-kit/CD31+ cells. By day 0, p>0.5. By day 1, * vs. other groups with different symbols (†, ‡, §), p<0.0001. By day 14, α vs. other groups with different symbols (ß, γ, κ), *p*<0.0001. (**E**) Illustrating the flow cytometric analysis of time courses of circulating number of Sca-1/CD31+ cells. By day 0, *p*>0.5. By day 1, * vs. other groups with different symbols (†, ‡, §), *p*<0.0001. By day 14, α vs. other groups with different symbols (ß, γ, κ), *p*<0.0001. (**F**) Illustrating the flow cytometric analysis of time courses of circulating number of KDR/CD34+ cells. By day 0, *p*>0.5. By day 1, * vs. other groups with different symbols (†, ‡, §), *p*<0.0001. By day 14, α vs. other groups with different symbols (ß, γ, κ), *p*<0.0001. (**G**) Illustrating the flow cytometric analysis of time courses of circulating number of VE-Cadherin/CD34+ cells. By day 0, *p*>0.5. By day 1, * vs. other groups with different symbols (†, ‡, §), *p*<0.0001. By day 14, α vs. other groups with different symbols (ß, γ, κ), *p*<0.0001. All statistical analyses were performed by one-way ANOVA, followed by Bonferroni multiple comparison post hoc test (n=6 for each group). Symbols (*, †, ‡, §) and (α, ß, γ, κ,) indicate significance (at 0.05 level). AHS = acute hemorrhagic stroke; F344^SC^ = sham control (^SC^) of Fischer344 (F344); DPP4^D-SC^ = sham control (^SC^) dipeptidyl peptidase 4 deficiency (DPP4^D^); F344^HS^ = hemorrhagic stroke (^HS^) in F344; DPP4^D-HS^ = hemorrhagic stroke (^HS^) in DPP4^D^; Sita = sitagliptin.

To elucidate the time courses of circulating levels of chemokines, ELISA analysis was utilized for the study. By day 0 prior to AHS procedure, the circulating levels of SDF-1α and GLP-1 were not significant difference among the five groups of the animals (refer to blue bar chart in Fig. 1A and 1B). However, by day 1 after AHS procedure, the circulating levels of SDF-1α and GLP-1 were significantly progressively increased from groups 1 (F344^SC^) and 2 (DPP4^D-SC^) to groups 3 (F344^HS^), 4 (F344^HS^ + Sita) and 5 (DPP4^D-HS^) (refer to red bar chart in Fig. 1A and 1B). Consistently, by day 14 after AHS procedure, these two parameters also exhibited an identical pattern (but less high) of day 3 among the five groups (refer to green bar chart in Fig. 1A and 1B).

By days 0, 1 and 14 prior to AHS procedure, the result of ELISA revealed that the circulating activity of DPP4 was not detected in group 2 animals (Fig. 1C). Additionally, this parameter was extremely low in groups 1 and 5 at these time points. Furthermore, this parameter also extremely low in groups 3 and 4 at day 0 prior to AHS induction (Fig. 1C).

By days 1 and 14 after AHS procedure, the circulating activities of DPP4 were lowest in groups 1 and 2, highest in group 3 and significantly increased in group 4 than in group 5 (Fig. 1C), suggesting that this extremely low parameter in circulation was able to be markedly increased in a manner of intrinsic response to ischemic stimulation.

Next, we utilized the flow cytometric analysis to measure the serial changes of circulatory EPCs among the five groups. As expected, by day 0, the circulating levels of C-kit/CD31+, Sca-1/CD31+, KDR/CD34+ and VE-Cadherin/CD34+ cells, four surface markers of EPCs, did not differ among the five groups (Fig. 1D-G). However, by days 1 and 14 after the AHS procedure, these parameters were significantly progressively increased from groups 1/2 to group 5, suggesting that both Sita therapy and DPP4^D^ genotype could regulate the circulating EPC levels in response to ischemic situation (Fig. 1D-G).

### The BIA and time courses of neurological status and BIV among five groups of animals

**Figure 2:**
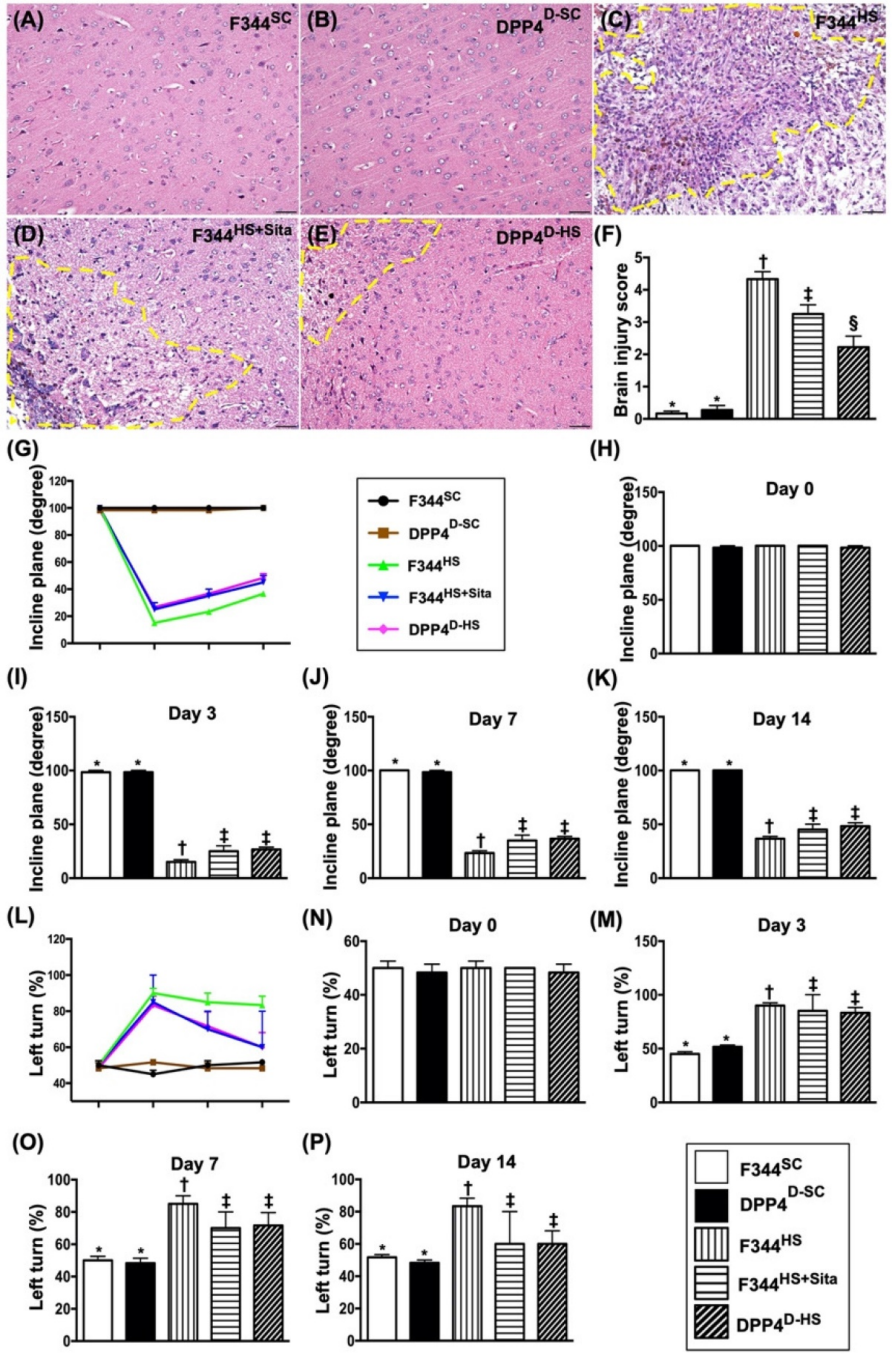

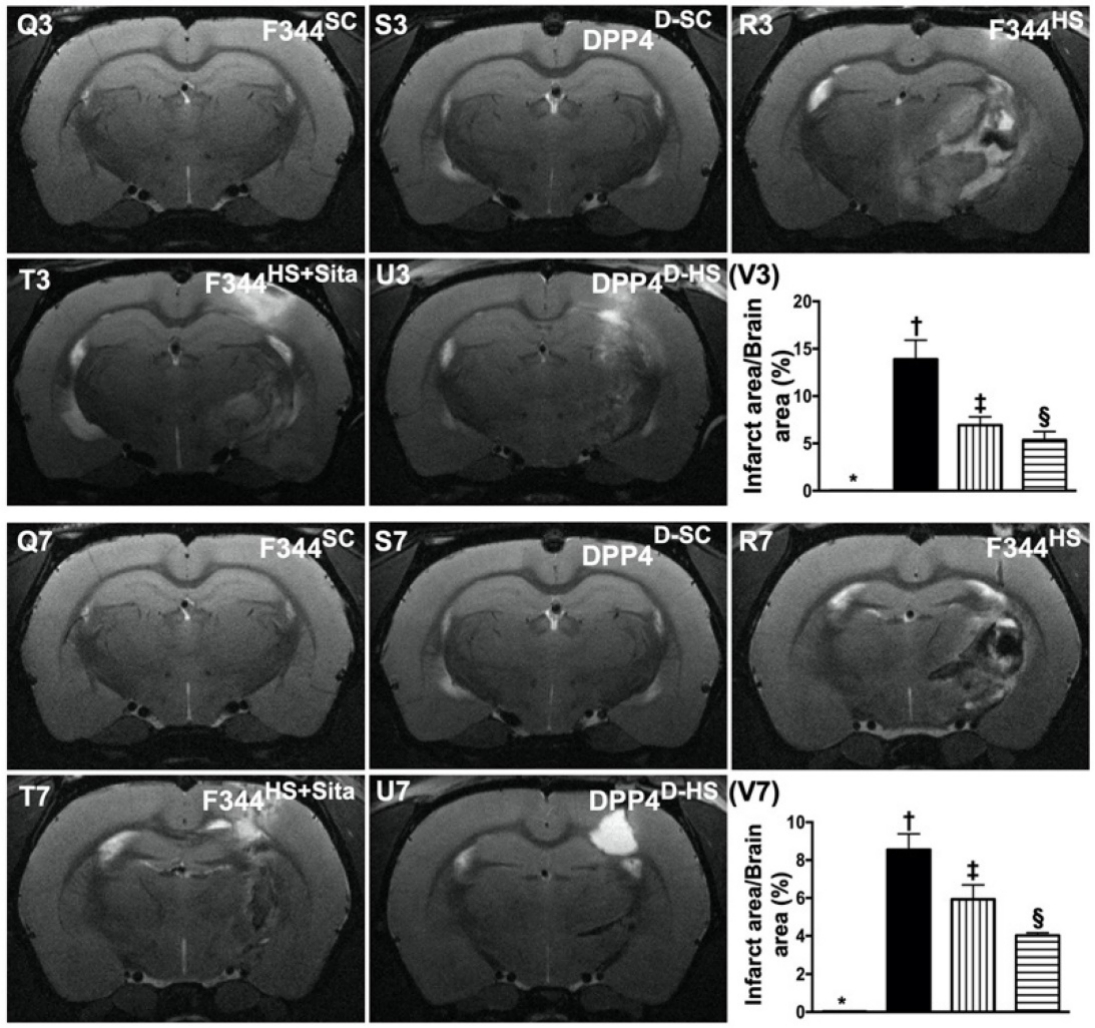
** The BIA and time courses of BIV and neurological status among five groups of animals.** (**A to E**) Illustrating the H.E stain (200x) for identification of BIA (gray color; the yellow dotted line indicates the boundary of BIA by day 14 after AHS procedure (n=4). (**F**) Statistical analysis of summated (three cross sections in each animal) BIA, * vs. other groups with different symbols (†, ‡, §), *p*<0.0001. (**G**) Illustrating the inclined plane test for determining limb motor function among days 0, 3, 7 and 14 after AHS procedure (n=6). (**H**) Statistical analysis by day 0, *p*>0.5. (**I**) Statistical analysis by day 3, * vs. other groups with different symbols (†, ‡), *p*<0.0001. (**J**) Statistical analysis by day 7, * vs. other groups with different symbols (†, ‡), *p*<0.0001. (**K**) Statistical analysis by day 14, * vs. other groups with different symbols (†, ‡), *p*<0.001. (**L**) Illustrating the corner test for determining neurological function among days 0, 3, 7 and 14 after AHS procedure (n=6). (**M**) Statistical analysis by day 0, *p*>0.5. (**N**) Statistical analysis by day 3, * vs. †, *p*<0.001. (**O**) Statistical analysis by day 7, * vs. other groups with different symbols (†, ‡), *p*<0.001. (**P**) Statistical analysis by day 14, * vs. other groups with different symbols (†, ‡), *p*<0.001. (**Q3 to U3**) Illustrating brain MRI by day 3 after AHS induction (white color) (n=4). (**V3**) Analysis of BHV, * vs. other groups with different symbols (†, ‡, §), *p*<0.0001. (**Q7 to U7**) Illustrating brain MRI by day 7 after AHS induction (white color) (n=4). (**V7**) Analysis of BIV, * vs. other groups with different symbols (†, ‡, §), *p*<0.001. All statistical analyses were performed by one-way ANOVA, followed by Bonferroni multiple comparison post hoc test. Symbols (*, †, ‡, §) indicate significance (at 0.05 level). AHS = acute hemorrhagic stroke; F344^SC^ = sham control (^SC^) of Fischer344 (F344); DPP4^D-SC^ = sham control (^SC^) dipeptidyl peptidase 4 deficiency (DPP4^D^); F344^HS^ = hemorrhagic stroke (^HS^) in F344; DPP4^D-HS^ = hemorrhagic stroke (^HS^) in DPP4^D^; Sita = sitagliptin, BIA = brain ischemic area; BIV = brain ischemic volume.

The H.E., stained brain specimen, was examined by microscope for determining the BIA in each group (Fig. 2A-E). The results showed that the BIA was absent in groups 1 and 2, highest in group 3 and significantly higher in group 4 than in group 5 (Fig. 2F).

The inclined plane test for determining limb motor function was conducted for each rat at baseline and on days 3, 7 and 14 after AHS induction (Fig. 2G). By day 0, this parameter did not differ among the five groups (Fig. 2H). However, by days 3, 7 and 14 this parameter was significantly lower in group 3 than in groups 1, 2, 4 and 5 and significantly lower in groups 4 and 5 than in groups 1 and 2, but it showed no difference between the former or between the latter two groups (Fig. 2I-K).

Next, the sensorimotor functional test (Corner test) was conducted for each rat at baseline and on days 3, 7 and 14 after AHS induction (Fig. 2L). By day 0, the neurological function did not differ among the five groups (Fig. 2N). However, the Corner test showed the attainment of a steady state of neurological functional impairment from days 3 to 14 following AHS procedure among the groups 3 to 5 animals (Fig. 2M-P). A significant improvement in neurological function was found apparently in groups 4 and 5 as compared with group 3 by day 7 after AHS (Fig. 2M-P). Further improvement by day 14 after AHS was observed in groups 4 and 5 but not in group 3 (Fig. 2M-P).

To elucidate the time courses of BIV, the brain MRI was performed for each group of animals prior to and by days 3 and 7 at acute phase of AHS (Fig. 2 Q3-U7). As expected, the baseline brain MRI showed no BIV observed among the five groups of animals. However, by days 3 and 7, the BIV was significantly increased in group 3 than in other groups, significantly increased in groups 4 and 5 than in groups 1 and 2 and significantly increased in group 4 than in group 5, but it showed no difference between groups 1 and 2 (Fig. 2 V3-V7).

### The protein expressions of inflammatory biomarkers in brain tissue by day 14 after AHS

**Figure 3:**
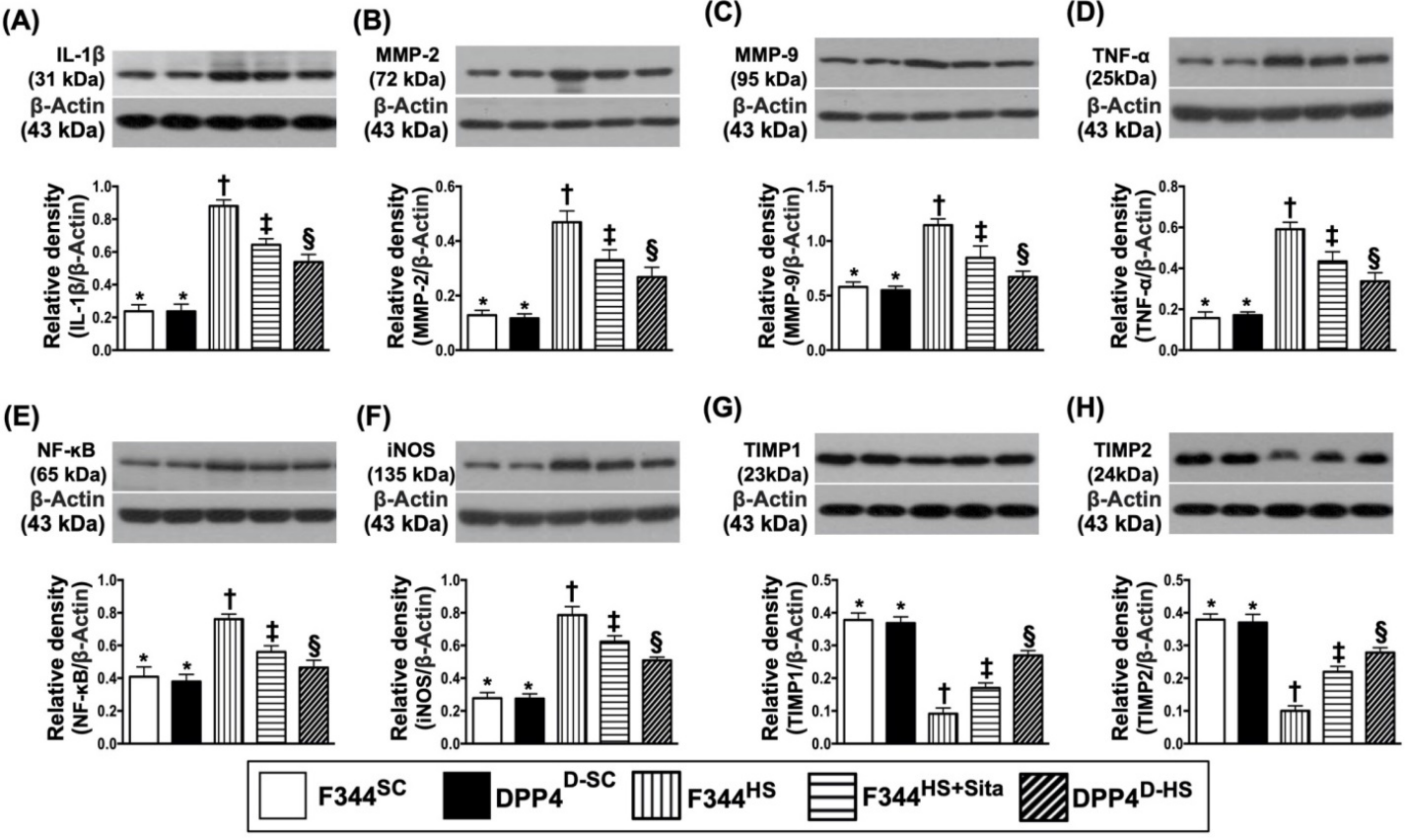
** The protein expressions of inflammatory biomarkers in brain tissue by day 14 after AHS.** (**A**) Protein expression of interleukin (IL)-1ß, * vs. other groups with different symbols (†, ‡, §), *p*<0.0001. (**B**) Protein expression of matrix metalloproteinase (MMP)-2, * vs. other groups with different symbols (†, ‡, §), *p*<0.0001. (**C**) Protein expression of MMP-9, * vs. other groups with different symbols (†, ‡, §), *p*<0.0001. (**D**) Protein expression of tumor necrosis factor (TNF)-α, * vs. other groups with different symbols (†, ‡, §), *p*<0.0001. (**E**) Protein expression of nuclear factor (NF)-κB, * vs. other groups with different symbols (†, ‡, §), *p*<0.0001. (**F**) Protein expression of induced nitric oxide synthase (iNOS), * vs. other groups with different symbols (†, ‡, §), *p*<0.0001. (**G**) Protein expression of tissue inhibitors of matrix metalloproteinase 1 (TIMP1), * vs. other groups with different symbols (†, ‡, §), *p*<0.0001. (**H**) Protein expression of TIMP2, * vs. other groups with different symbols (†, ‡, §), *p*<0.0001. All statistical analyses were performed by one-way ANOVA, followed by Bonferroni multiple comparison post hoc test (n=6 for each group). Symbols (*, †, ‡, §) indicate significance (at 0.05 level). AHS = acute hemorrhagic stroke; F344^SC^ = sham control (^SC^) of Fischer344 (F344); DPP4^D-SC^ = sham control (^SC^) dipeptidyl peptidase 4 deficiency (DPP4^D^); F344^HS^ = hemorrhagic stroke (^HS^) in F344; DPP4^D-HS^ = hemorrhagic stroke (^HS^) in DPP4^D^; Sita = sitagliptin.

The protein expressions of IL-1ß, MMP-2, MMP-9, TNF-α, NF-κB, and iNOS, six indicators of inflammation, were highest in group 3 and lowest in groups 1 and 2, and significantly higher in group 4 than in group 5, but no difference between groups 1 and 2 (Fig. 3A-F). On the other hand, the protein expressions of TIMP1 and TIMP2, two indicators of metalloproteinase inhibitors, displayed an opposite pattern of inflammation among the five groups (Fig. 3G & H).

### Protein expressions of apoptotic, fibrotic, oxidative stress, GLP-1R, SDF-1 and cell stress signaling pathway biomarkers in brain tissue by day 14 after AHS

**Figure 4:**
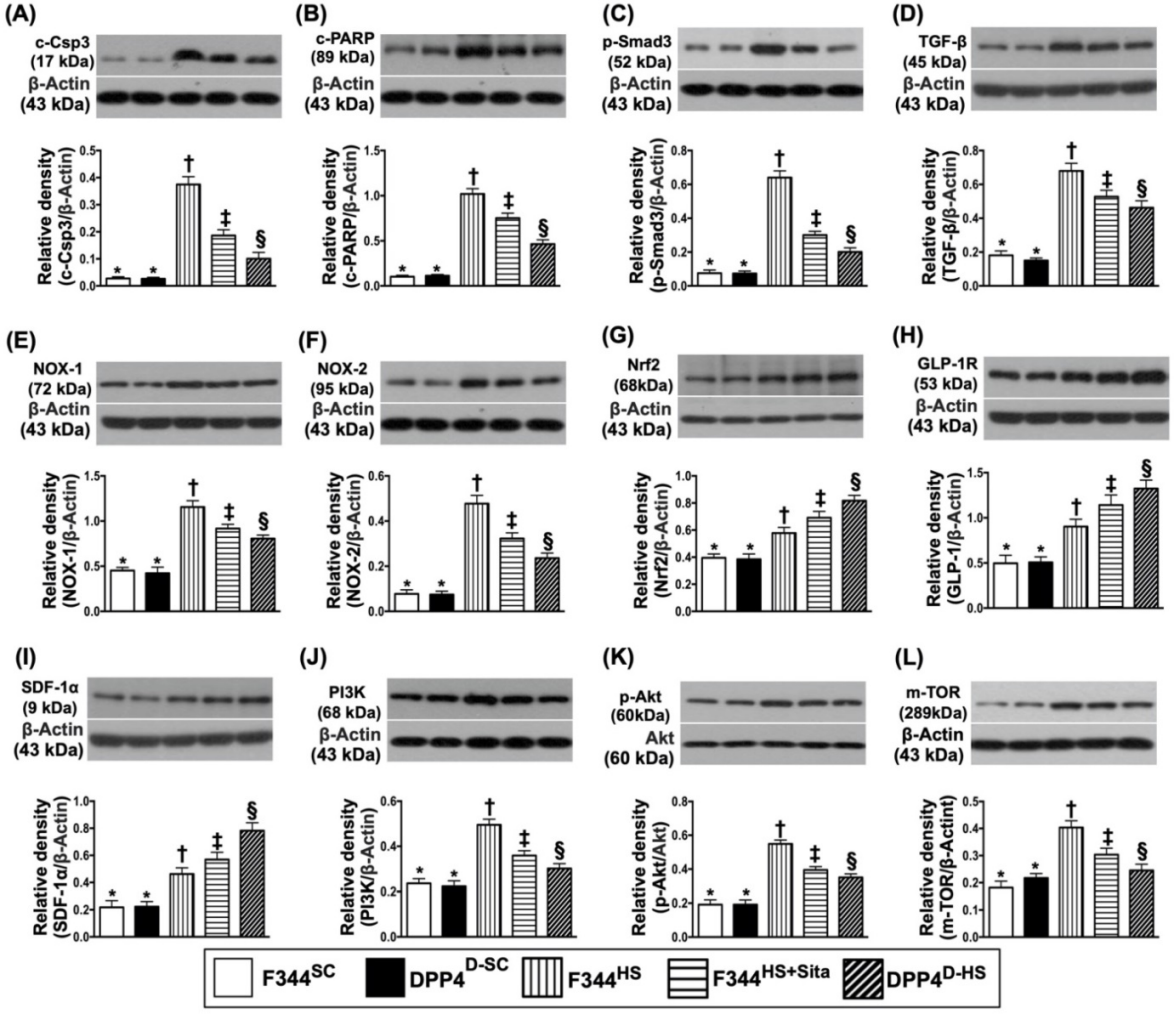
** Protein expressions of apoptotic, fibrotic and oxidative stress, GLP-1R, SDF-1 biomarkers and cell stress signaling pathway in brain tissue by day 14 after AHS.** (**A**) Protein expression of cleaved caspase 3 (c-Csp3), * vs. other groups with different symbols (†, ‡, §), *p*<0.0001. (**B**) Protein expression of cleaved Poly (ADP-ribose) polymerase (c-PARP), * vs. other groups with different symbols (†, ‡, §), *p*<0.0001. (**C**) Protein expression of Smad3, * vs. other groups with different symbols (†, ‡, §), *p*<0.0001. (**D**) Protein expression of transforming growth factor (TGF)-ß, * vs. other groups with different symbols (†, ‡, §), *p*<0.0001. (**E**) Protein expression of NOX-1, * vs. other groups with different symbols (†, ‡, §), *p*<0.0001. (**F**) Protein expression of NOX-2, * vs. other groups with different symbols (†, ‡, §), *p*<0.0001. (**G**) Protein expression of nuclear factor erythroid 2-related factor 2 (Nrf2), * vs. other groups with different symbols (†, ‡, §), *p*<0.0001. (**H**) Protein expression of glucagon-like peptide 1 receptor (GLP-1R), * vs. other groups with different symbols (†, ‡, §), *p*<0.0001. (**I**) Protein expression of stromal cell-derived factor (SDF)-1α, * vs. other groups with different symbols (†, ‡, §), *p*<0.0001. (**J**) Protein expression of PI3K, * vs. other groups with different symbols (†, ‡, §), *p*<0.0001. (**K**) Protein expression of phosphorylated (p)-Akt, * vs. other groups with different symbols (†, ‡, §), *p*<0.0001. (**L**) Protein expression of m-TOR, * vs. other groups with different symbols (†, ‡, §), *p*<0.0001. All statistical analyses were performed by one-way ANOVA, followed by Bonferroni multiple comparison post hoc test (n=6 for each group). Symbols (*, †, ‡, §) indicate significance (at 0.05 level). AHS = acute hemorrhagic stroke; F344^SC^ = sham control (^SC^) of Fischer344 (F344); DPP4^D-SC^ = sham control (^SC^) dipeptidyl peptidase 4 deficiency (DPP4^D^); F344^HS^ = hemorrhagic stroke (^HS^) in F344; DPP4^D-HS^ = hemorrhagic stroke (^HS^) in DPP4^D^; Sita = sitagliptin.

Western blotting was performed for determining the apoptotic, fibrotic and oxidative stress biomarkers in hemorrhagic brain tissue. As expected, the protein expressions of cleaved caspase 3 and cleaved PARP, two indicators of apoptosis, and the protein expressions of Smad3 and TGF-ß, two indicators of fibrosis, were highest in group 3 and lowest in groups 1 and 2, and significantly higher in group 4 than in group 5, but no difference between groups 1 and 2 (Fig. 4A-D). Consistently, the protein expressions of NOX-1 and NOX-2, two indices of oxidative stress, displayed a similar pattern of apoptosis among the five groups (Fig. 4E & F). On the other hand, the protein expression of Nrf2, an indicator of antioxidant, exhibited an opposite pattern of oxidative stress (Fig. 4G).

Next, we performed the Western blot for elucidating the role of DPP4^D^/Sita on regulating the bioactivity of GLP-1R and SDF-1 (Fig. 4H & I). The results showed that the protein expressions of these two parameters were significantly progressively increased from groups 1 and 2 to group 5, suggesting an intrinsic response to ischemic stimulation that was further augmented by DPP4^D^ and Sita therapy (Fig. 4H & I).

To further assess the cell stress signaling in rat after AHS, the Western blotting tool was utilized in the present study. The result showed that the protein expressions of PI3K, P-Akt and m-TOR were highest in group 3, lowest in groups 1 and 2, and significantly increased in group 4 than in group 5, but they did not differ between groups 1 and 2 (Fig. 4J-L). These findings implicated a typical apoptosis/cell death signaling that occurred in setting of AHS.

### Inflammatory cellular expressions in brain tissue by day 14 after AHS

**Figure 5:**
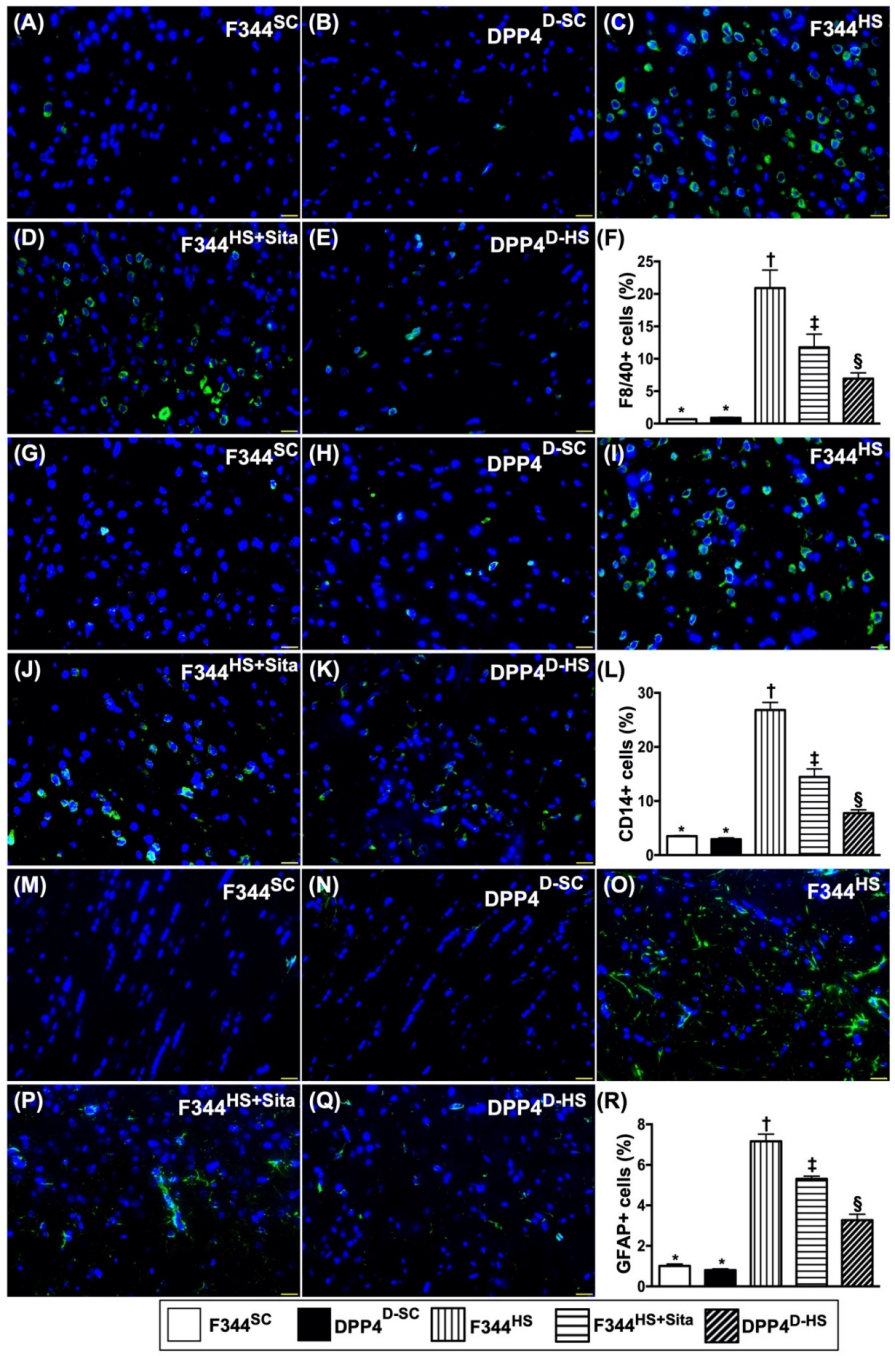
** Inflammatory cellular expressions in brain tissue by day 14 after AHS.** (**A to E**) Illustrating the immunofluorescent (IF) microscopic finding (400x) for identification of F4/80+ cells (green color). F) Analytical result of number of F4/80+ cells, * vs. other groups with different symbols (†, ‡, §), *p*<0.0001. (**G to K**) Illustrating the IF microscopic finding (400x) for identification of CD14+ cells (green color). (**L**) Analytical result of number of CD14+ cells, * vs. other groups with different symbols (†, ‡, §), *p*<0.0001. (**M to Q**) Illustrating the IF microscopic finding (400x) for identification of glial fibrillary acidic protein (GFAP)+ cells (green color). (**R**) Analytical result of number of GFAP+ cells, * vs. other groups with different symbols (†, ‡, §), *p*<0.0001. All statistical analyses were performed by one-way ANOVA, followed by Bonferroni multiple comparison post hoc test (n=6 for each group). Symbols (*, †, ‡, §) indicate significance (at 0.05 level). AHS = acute hemorrhagic stroke; F344^SC^ = sham control (^SC^) of Fischer344 (F344); DPP4^D-SC^ = sham control (^SC^) dipeptidyl peptidase 4 deficiency (DPP4^D^); F344^HS^ = hemorrhagic stroke (^HS^) in F344; DPP4^D-HS^ = hemorrhagic stroke (^HS^) in DPP4^D^; Sita = sitagliptin.

We utilized the IF microscopic examination to identify the role of DPP4^D^/Sita on regulating the inflammatory cell expressions in hemorrhagic area. As expected of our original hypothesis, the cellular expressions of F4/80+ (Fig. 5A-E), CD14+ (Fig. 5G-K) and GFPA+ cells (Fig. 5M-Q), three indicators of inflammation, were highest in group 3, lowest in groups 1 and 2, and significantly higher in group 4 than in group 5 (Fig. 5-F, L & R).

### Cellular expressions of angiogenesis biomarkers in brain tissue by day 14 after AHS

**Figure 6:**
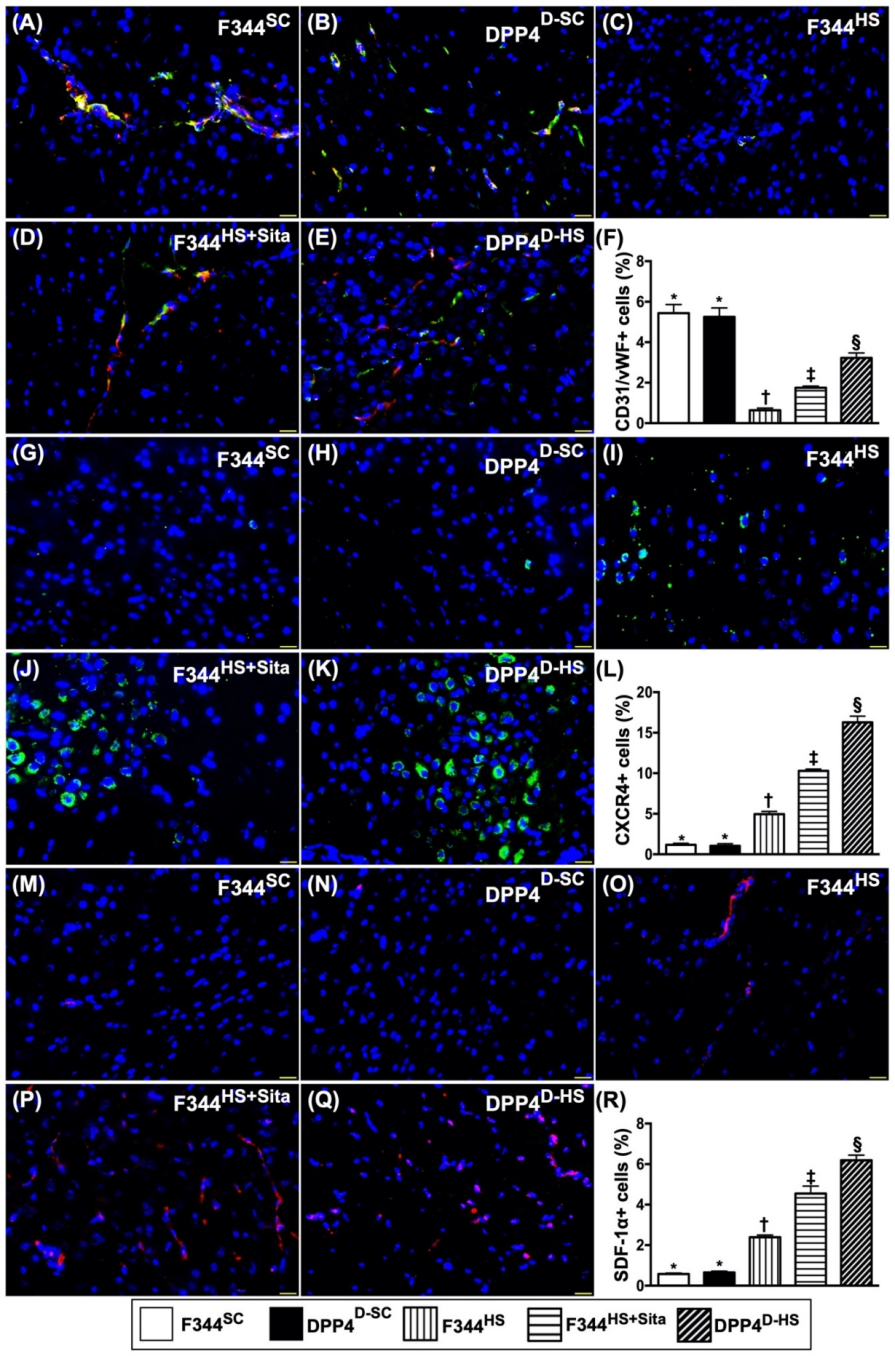
** Cellular expressions of angiogenesis biomarkers in brain tissue by day 14 after AHS.** (**A to E**) Illustrating the immunofluorescent (IF) microscopic finding (400x) for identification of doubly positively stained CD31/vWF cells (i.e., yellow-green color. green color indicated CD31 staining; red color indicated vWF staining). (**F**) Analytical result of number of CD31/vWF+ cells, * vs. other groups with different symbols (†, ‡, §), *p*<0.0001. (**G to K**) Illustrating the IF microscopic finding (400x) for identification of CXCR4+ cells (green color). (**L**) Analytical result of number of CXCR4+ cells, * vs. other groups with different symbols (†, ‡, §), *p*<0.0001. (**M to Q**) Illustrating the IF microscopic finding (400x) for identification of stromal cell-derived factor (SDF)-1α+ cells (green color). (**R**) Analytical result of number of SDF-1α+ cells, * vs. other groups with different symbols (†, ‡, §), *p*<0.0001. All statistical analyses were performed by one-way ANOVA, followed by Bonferroni multiple comparison post hoc test (n=6 for each group). Symbols (*, †, ‡, §) indicate significance (at 0.05 level). AHS = acute hemorrhagic stroke; F344^SC^ = sham control (^SC^) of Fischer344 (F344); DPP4^D-SC^ = sham control (^SC^) dipeptidyl peptidase 4 deficiency (DPP4^D^); F344^HS^ = hemorrhagic stroke (^HS^) in F344; DPP4^D-HS^ = hemorrhagic stroke (^HS^) in DPP4^D^; Sita = sitagliptin.

The microscopic findings revealed that the number of doubly positively stained CD31/vWF cells, an indicator of integrity of endothelial cells, was highest in groups 1 and 2 and progressively increased from groups 3 to 5 (Fig. 6A-F). Additionally, the cellular expressions of CXCR4 (Fig. 6G-K) and SDF-1α (Fig. 6M-Q), two indicators of angiogenesis, were identified to significantly progressively increased from groups 1/2 to group 5 (Fig. 6L & R), highlighting an intrinsic response to ischemic stimulation and enhanced by DPP4^D^ and Sita stimulation.

### Cellular expressions of DNA-damaged biomarkers in brain tissue by day 14 after AHS

**Figure 7:**
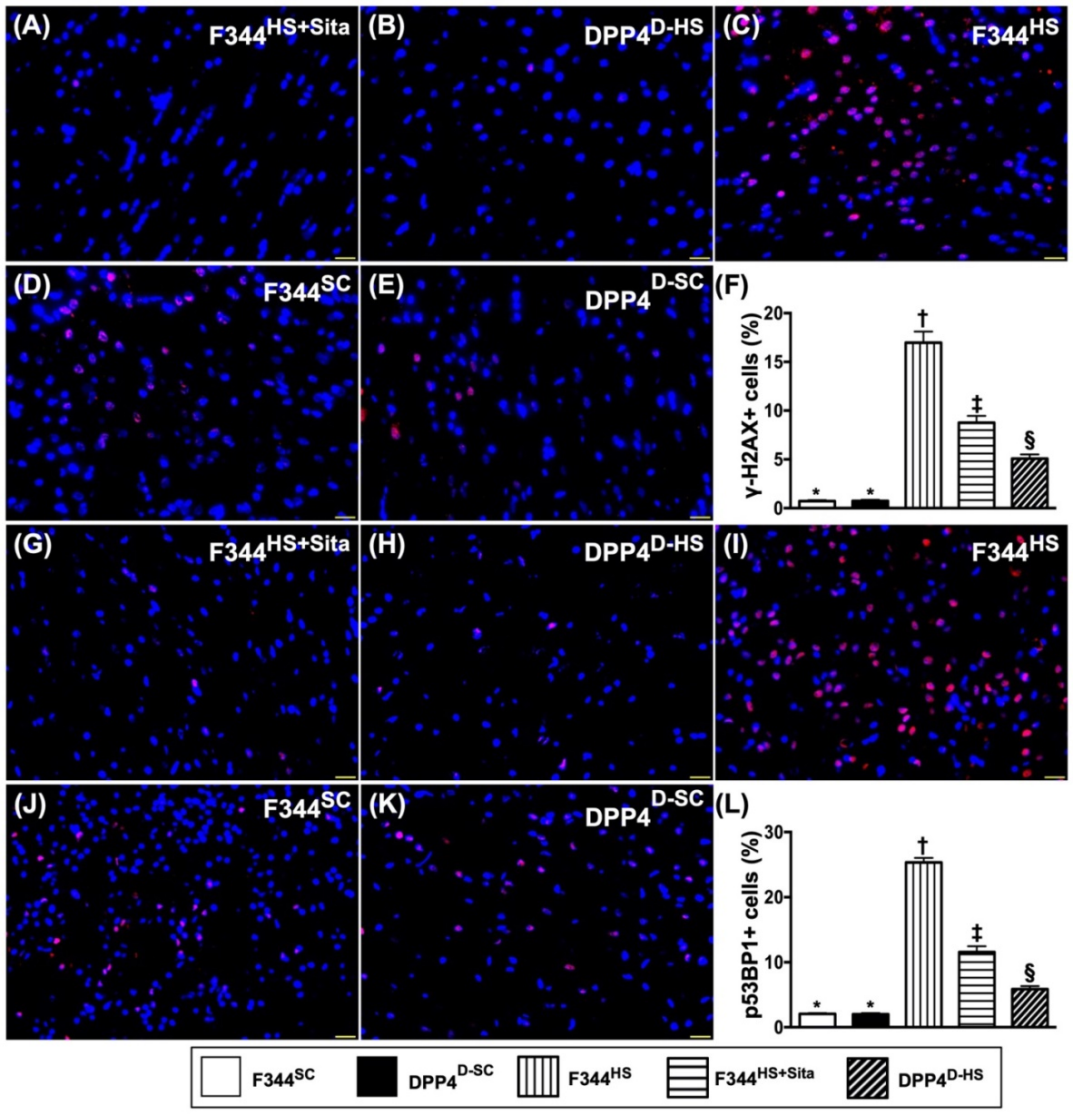
** Cellular expressions of DNA-damaged biomarkers in brain tissue by day 14 after AHS.** (**A to E**) Illustrating the immunofluorescent (IF) microscopic finding (400x) for identification of doubly positively stained γ-H2AX cells (pink color). (**F**) Analytical result of number of γ-H2AX+ cells, * vs. other groups with different symbols (†, ‡, §), *p*<0.0001. (**G to K**) Illustrating the IF microscopic finding (400x) for identification of p53BP1+ cells (green color). (**L**) Analytical result of number of p53BP1+ cells, * vs. other groups with different symbols (†, ‡, §), *p*<0.0001. All statistical analyses were performed by one-way ANOVA, followed by Bonferroni multiple comparison post hoc test (n=6 for each group). Symbols (*, †, ‡, §) indicate significance (at 0.05 level). AHS = acute hemorrhagic stroke; F344^SC^ = sham control (^SC^) of Fischer344 (F344); DPP4^D-SC^ = sham control (^SC^) dipeptidyl peptidase 4 deficiency (DPP4^D^); F344^HS^ = hemorrhagic stroke (^HS^) in F344; DPP4^D-HS^ = hemorrhagic stroke (^HS^) in DPP4^D^; Sita = sitagliptin.

Further, we utilized the IF microscope to clarify the role of DPP4^D^/Sita on attenuating the DNA damage in brain after AHS. Undoubtedly, the cellular expressions of γ-H2AX+ (Fig. 7A-E) and p53BP1+ (Fig. 7G-K) cells, two cellular levels of DNA-damaged biomarkers, were highest in group 3, lowest in groups 1 and 2, significantly higher in group 4 than in group 5, but there was no difference between groups 1 and 2 (Fig. 7F & L).

### Sitagliptin protected the cell proliferation and against apoptosis undergoing oxidative stress through PI3K/p-AKT/m-TOR

To elucidate the protective effect of sitagliptin (i.e., the specificity of DDP4 inhibitor) on N2a cells, the experimental study was categorized into four groups: Na2 cell only (i.e., control group) (group I), N2a cell treated by H_2_O_2_ (group II), N2a cell treated by H_2_O_2_ and sitagliptin (group III), N2a cell + H_2_O_2_ + sitagliptin + LY-294002 (i.e., a specific PI3K inhibitor) (group IV).

**Figure 8:**
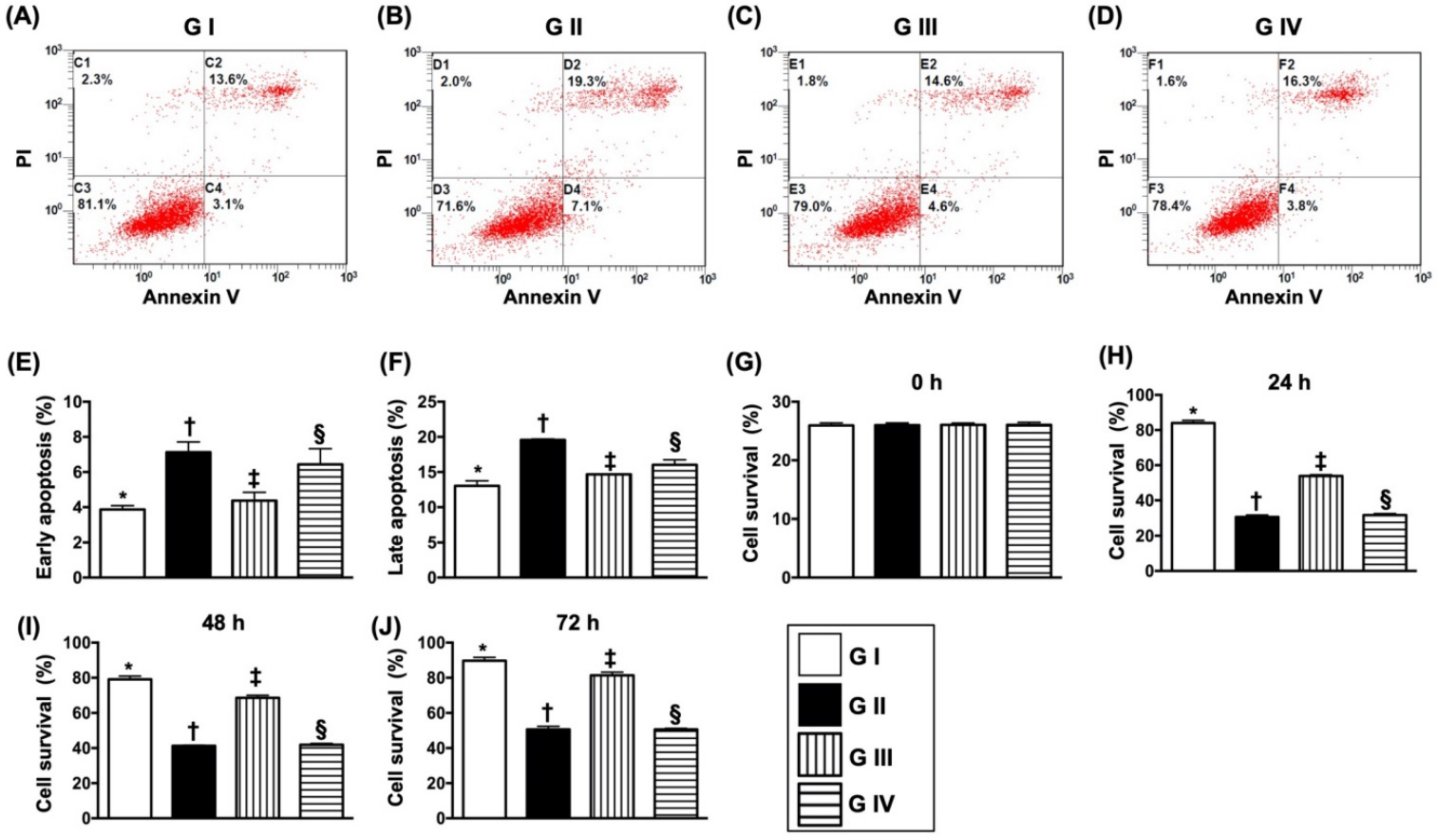
** Sitagliptin preserved the cell survival rate and against cellular apoptosis *in situ*ation of oxidative stress.** (**A-D**) Illustrating the early (AN-V+/PI-) and late (AN-V+/PI+) N2a cell apoptosis. (**E**) The percentage of early apoptosis, * vs. other groups with different symbols (†, ‡, §), *p*<0.0001. (**F**) The percentage of early apoptosis, * vs. other groups with different symbols (†, ‡, §), *p*<0.0001. (**G**) The percentage of late apoptosis, * vs. other groups with different symbols (†, ‡, §), *p*<0.0001. (**H**) MTT assay for cell survival rate at 0 h, *p*<0.5. (**I**) MTT assay for cell survival rate at 24 h, * vs. other groups with different symbols (†, ‡, §), *p*<0.0001. (**J**) MTT assay for cell survival rate at 48 h, * vs. other groups with different symbols (†, ‡, §), *p*<0.0001. (**K**) MTT assay for cell survival rate at 72 h, * vs. other groups with different symbols (†, ‡, §), *p*<0.0001. All statistical analyses were performed by one-way ANOVA, followed by Bonferroni multiple comparison post hoc test (n=4 for each group). Symbols (*, †, ‡, §) indicate significance (at 0.05 level). Group I = Na2 cell only (i.e., control group); Group II = N2a cell treated by H2O2; Group III = N2a cell treated by H^2^O^2^ and sitagliptin; Group IV = N2a cell + H^2^O^2^ + sitagliptin + LY-294002.

The flow cytometric analysis (Fig. 8A-D) demonstrated that early (AN-V+/PI-) and late (AN-V+/PI+) N2a cells were highest in group II, lowest in group I and significantly lower in group III than in group IV (Fig. 8E & F). Additionally, the MTT assay showed that the Na2 cell proliferation/survival rate just expressed an opposite pattern of apoptosis by the time points of 24, 48 and 72 h (Fig. 8G-J).

**Figure 9:**
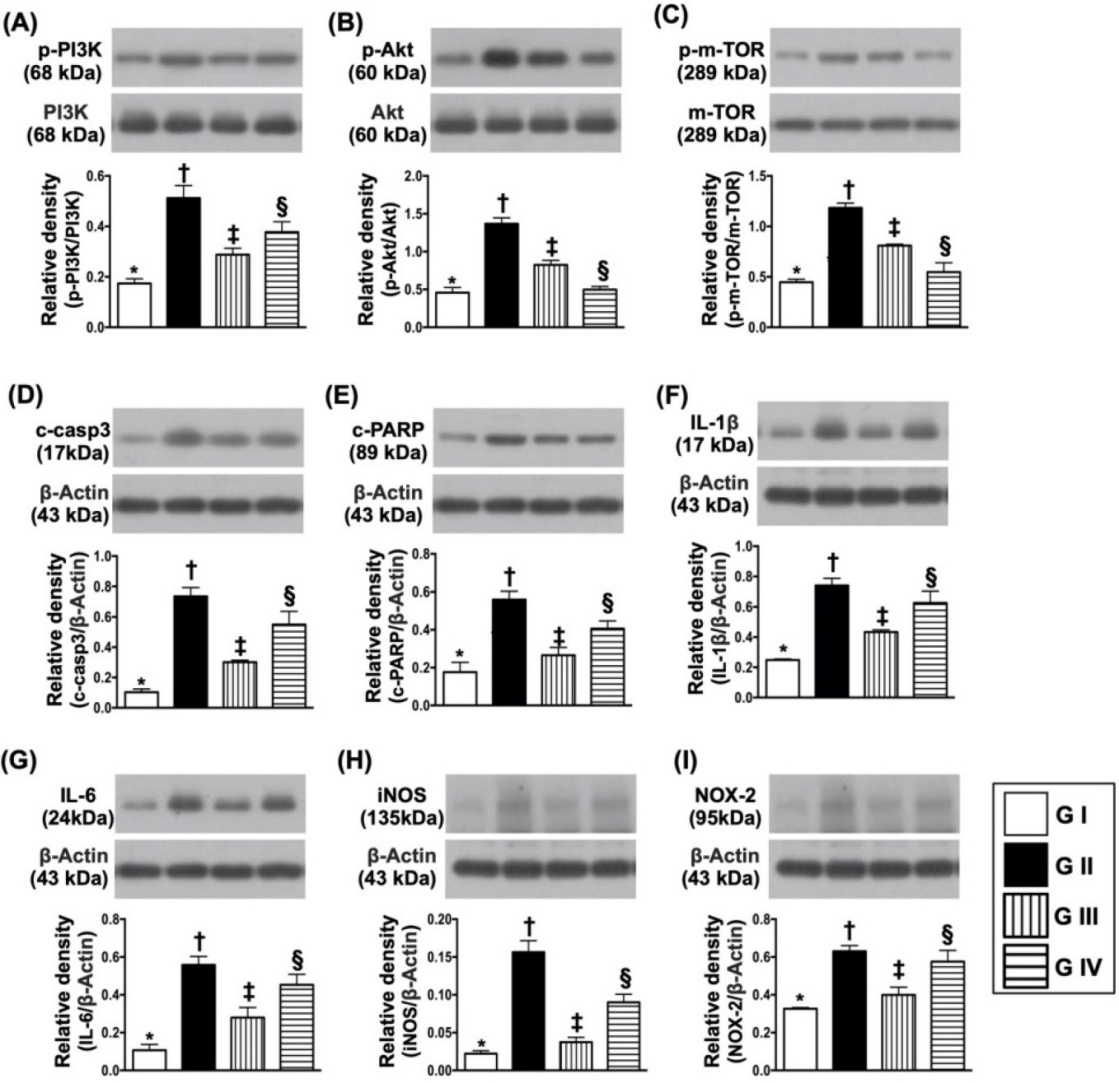
** Protein expressions of cell survival/death signaling, oxidative-stress and inflammatory biomarkers.** (**A**) Protein expression of phosphorylated (p)-PI3K, * vs. other groups with different symbols (†, ‡, §), *p*<0.001. (**B**) Protein expression of p-AKT, * vs. other groups with different symbols (†, ‡, §), *p*<0.0001. (**C**) Protein expressions of p-m-TOR* vs. other groups with different symbols (†, ‡, §), *p*<0.0001. (**D**) Protein expression of cleaved caspase 3 (c-casp3), * vs. other groups with different symbols (†, ‡, §), *p*<0.0001. (**E**) Protein expression of c-PARP, * vs. other groups with different symbols (†, ‡, §), *p*<0.001. (**F**) Protein expression of interleukin (IL)-1ß, * vs. other groups with different symbols (†, ‡, §), *p*<0.0001. (**G**) Protein expression of IL-6, * vs. other groups with different symbols (†, ‡, §), *p*<0.001. (**H**) Protein expression of inducible nitric oxide synthase (iNOS), * vs. other groups with different symbols (†, ‡, §), *p*<0.0001. (**I**) Protein expression of NOX-2, * vs. other groups with different symbols (†, ‡, §), *p*<0.003. All statistical analyses were performed by one-way ANOVA, followed by Bonferroni multiple comparison post hoc test (n=4 for each group). Symbols (*, †, ‡, §) indicate significance (at 0.05 level). Group I = Na2 cell only (i.e., control group); Group II = N2a cell treated by H^2^O^2^; Group III = N2a cell treated by H^2^O^2^ and sitagliptin; Group IV = N2a cell + H^2^O^2^ + sitagliptin + LY-294002.

**Figure 10:**
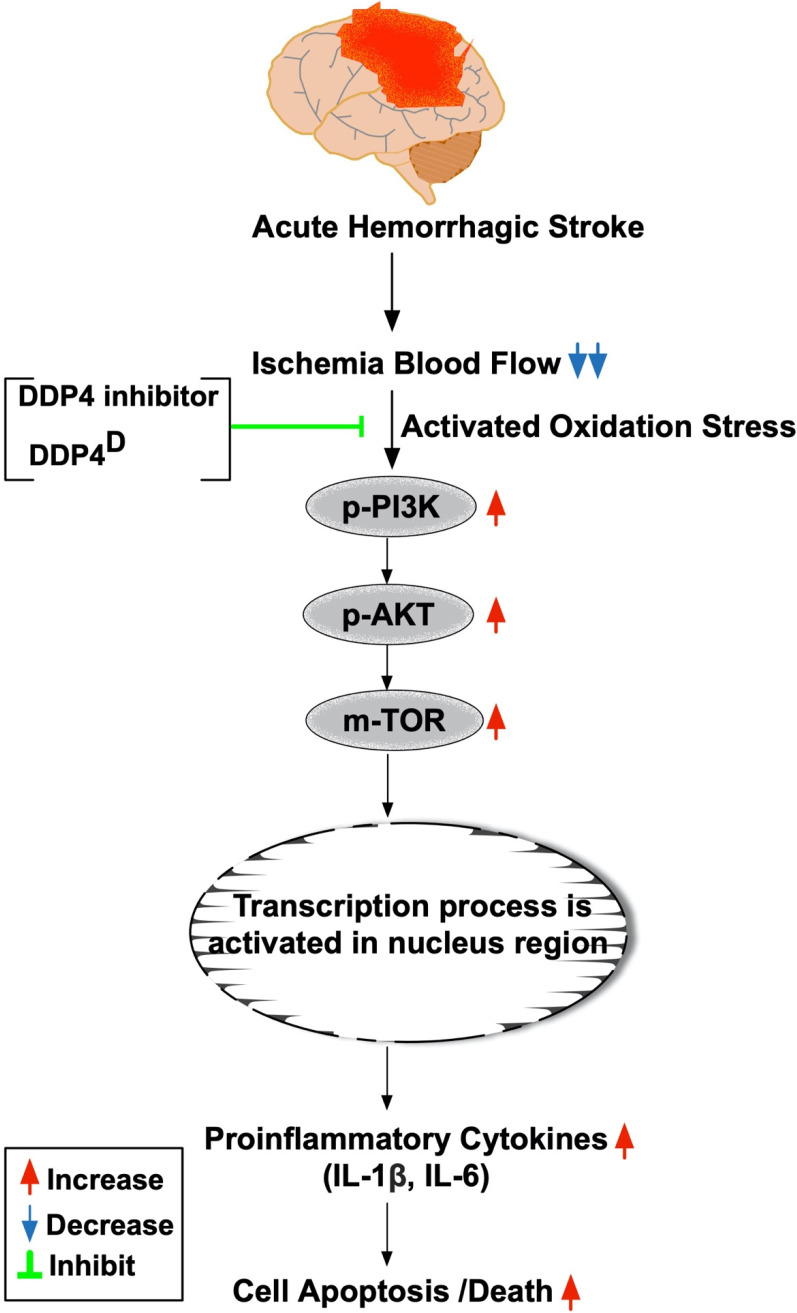
The prosed schematic proposed mechanism underlying the impact of DDP4 inhibition on protecting the cell from apoptosis and oxidative stress through regulating the PI3K/AKT/m-TOR signaling pathway. AHS = acute hemorrhagic stroke; DDP4^D^ = DDP4 deficiency; IL= interleukin.

To further clarify whether sitagliptin treatment on protecting the cells against oxidative stress damage and apoptosis was through downregulating the PI3K/AKT/m-TOR signaling pathway, Western blot analysis for the protein levels of these biomarkers were also performed. The results showed that the protein expressions of p-PI3K, p-AKT and m-TOR exhibited an identical pattern of MTT assay (Fig. 9A-C). Additionally, the protein expressions of induced nitric oxide synthase (iNOS) and NOX2, two oxidative-stress indicators (Fig. 9D & E), protein expressions of cleaved caspase 3 and cleaved PARP, two indicators of apoptosis and protein expressions of IL-1ß and IL-6, two indicators of proinflammatory cytokines, also exhibited an identical pattern of MTT assay. These *in vitro* findings implicated that sitagliptin protected the Na2 cells against oxidative stress damage could mainly through PI3K and its down-stream signaling (i.e., AKT/m-TOR) (refer to Fig. 10).

## Discussion

This study which investigated the impact of abrogating the DPP4 enzyme activity (i.e., by utilizing DPP4 mutant rat or Sita) on protecting the brain and the corresponding functional integrity against the AHS yielded several preclinical striking implications. First, Sita was comparable with DPP4^D^ on reducing the BHV/BHA and preserving the neurological function in AHS rat. Second, as compared with AHS animals, the circulating levels of chemokines (i.e., SDF-1α and GLP-1) and EPCs (i.e., C-kit/CD31+, Sca-1/CD31+, KDR/CD34+ and VE-Cadherin/CD34+) were remarkably increased in DPP4^D^ and Sita-treated animals, suggesting these results were mainly due to the suppression of DPP4 enzyme activity. Third, the underlying mechanism of DPP4^D^ and Sita treatment effectively preserving the brain architecture and the integrity of neurological function in setting of AHS was identified mainly due to downregulations of the inflammatory, oxidative-stress and cell stress signaling.

The most important finding in the present study was that the BHV (i.e., by brain MRI finding at days 2 and 7) and BHA (i.e., by histopathological finding at day 14) were significantly reduced in DPP4^D-HS^ and F344^HS^ + Sita animals than in F344^HS^ counterparts after AHS. Of particularly important finding in the present study was that the neurological function was significantly preserved in DPP4^D-HS^ and F344^HS^ + Sita animals than in F344^HS^ counterparts by days 3, 7 and 14 after AHS. These findings highlight that the DPP4 inhibitors may play an importantly accessory role for those of AHS patients.

We have previously identified that Sita (i.e., an DPP4 inhibitor) therapy significantly suppressed the DPP4 activity in circulation which, in turn, markedly increased circulating and tissue levels of SDF-1α and GLP-1 in setting of organ ischemia in rodent 29-32. Additionally, our studies have further revealed that Sita therapy significantly protected the organs against ischemia-caused damage 29-32. Intriguingly, we have further revealed that the underlying mechanisms involved in protecting the organ from ischemia after Sita therapy were mainly through suppressing the inflammation, oxidative stress, apoptosis, fibrosis and DNA damage 29-32. An essential finding in the present study was that as compared with F344^HS^, the circulating levels of SDF-1α and GLP-1 were substantially increased, whereas the circulatory activity of DPP4 was remarkably reduced in DPP4^D-HS^ and F344^HS^ + Sita animals. Additionally, the molecular-cellular levels of inflammatory, oxidative-stress, apoptotic, fibrotic and DNA-damaged biomarkers were consistently markedly suppressed DPP4^D-HS^ and F344^HS^ + Sita animals. Accordingly, our findings, in addition to corroborating with the results of previous studies 29-32, could partially explain why the BIA was notably reduced, and the neurological function was significantly preserved in DPP4^D-HS^ and F344^HS^ + Sita animals than those of F344^HS^ counterparts.

It is well known that SDF-1α is an extremely important chemokine for EPC mobilization from bone to circulation and EPC homing from circulation to ischemic area 33-36. Additionally, studies have further demonstrated that an increased SDF-1α level in ischemic area played a crucial role for EPC homing to the ischemic area for angiogenesis and neovascularization 35, 36. A principal finding in the present study was that the circulating levels of EPCs and the protein expressions of SDF-1α and GLP-1R in ischemic region were significantly increased in DPP4^D-HS^ and F344^HS^ + Sita groups than in F344^HS^ counterpart. Our finding, in addition to being supported by those of previous studies 29-36, could explain why the cellular level of angiogenesis was significantly increased in ischemic zone in DPP4^D-HS^ and F344^HS^ + Sita groups than in F344^HS^ group. Our finding could further explain the significant preservation of brain architecture and neurological integrity in DPP4^D-HS^ and F344^HS^ + Sita groups could be, at least in part, due to angiogenesis and restoration of the blood flow in ischemic area.

It is well recognized that intrinsic cellular responses are always elicited in response to stress stimulation for survival. Our previous study has shown that MAPK and Akt signaling markedly upregulated in acute myocardial infarction in porcine that was suppressed by tacrolimus treatment.^37^ Interestingly, in the *in vivo* studies, we also found that the PI3K/p-AKT/m-TOR signaling pathway was markedly upregulated in F344^HS^ animals than in SC counterparts, suggesting an intrinsic response to ischemic stimulation. However, this signaling pathway was significantly attenuated in DPP4^D-HS^ and F344^HS^ + Sita animals. Additionally, in the present *in vitro* study, we found that PI3K/p-AKT/m-TOR signaling pathway was also remarkedly upregulated by oxidative stress that was significantly downregulated by sitagliptin therapy. Accordingly, our *in vitro* results supported our *in vivo* findings and highlighted that inhibiting the DPP4 activity would rescue the cells from apoptosis and death.

## Study limitations

**Figure 11:**
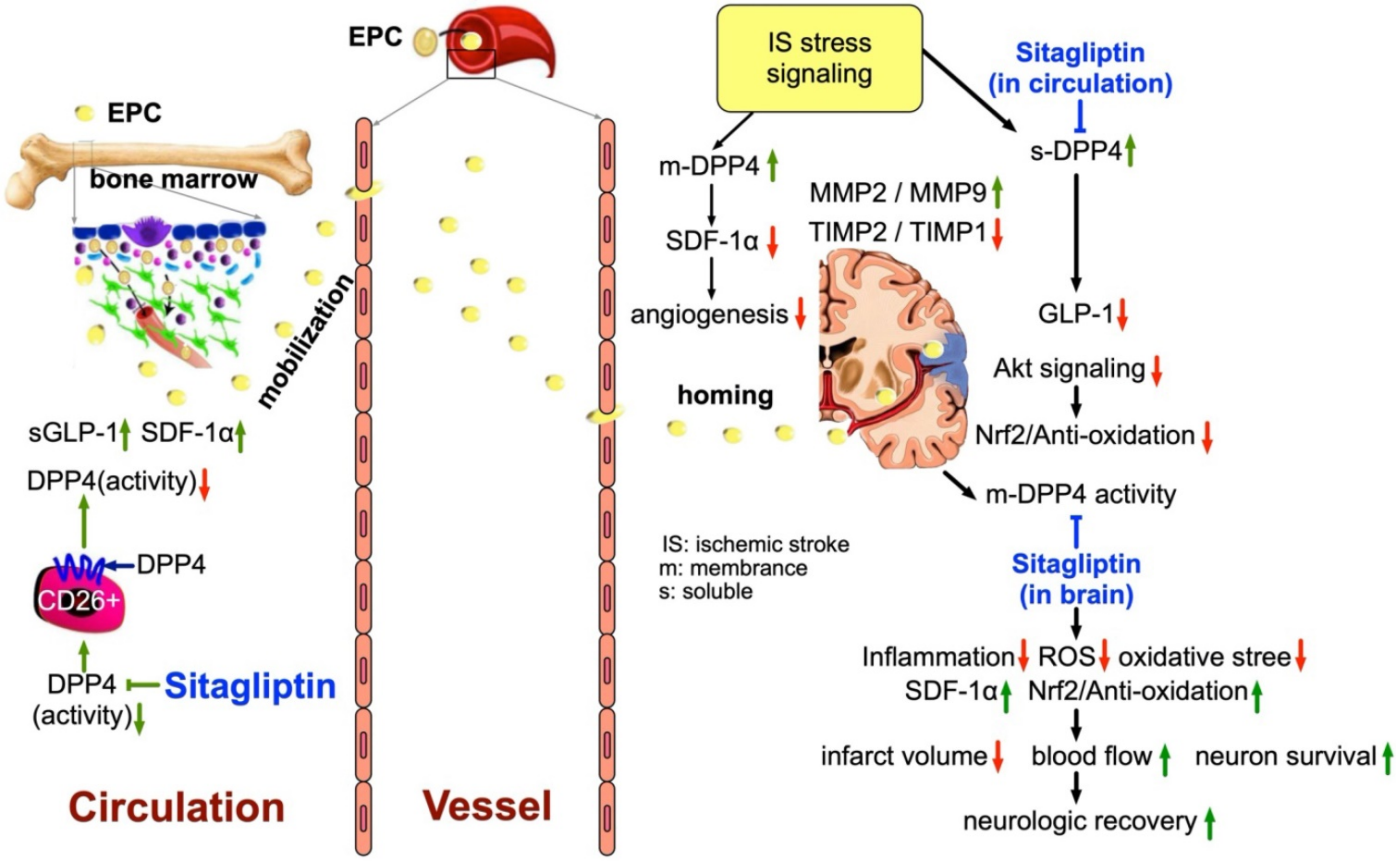
** The schematic proposed mechanism underlying the impact of DDP4 inhibition on protecting the brain architecture and neurological function against acute hemorrhagic stroke injury.** EPC = endothelial progenitor cell; SDF = stromal cell-derived factor; GLP = glucagon-like peptide; DDP4 = dipeptidyl peptidase 4; MMP = matrix metalloproteinase; TIMP = tissue inhibitors of matrix metalloproteinase.

This study has several limitations; firstly, the study period was only 14 days. Accordingly, despite the Sita therapy was promising and attractive in terms of short-term effect the long-term outcome of this therapy remains unclear. Second, this study did not provide a diabetic group; therefore, whether Sita therapy also is effective on protecting the brain in setting of AHS diabetic rat is still uncertain. Third, the exactly underlying mechanism of DDP4 inhibitor on protecting the brain from AHS may be more complicated than our findings. Figure 11 schematically illustrated the mechanism underlying the impact of DDP4 inhibition on protecting the brain architecture and neurological function against AHS injury.

In conclusion, the results of the present study demonstrated that Sita therapy which is utilized in our daily clinical practice was found to be consistent with DPP4^D^ on protecting the brain architecture and neurological function against AHS damage.
